# Dissection of the role of a Src homology 3 domain in the evolution of binding preference of paralogous proteins

**DOI:** 10.1093/genetics/iyad175

**Published:** 2023-10-04

**Authors:** Pascale Lemieux, David Bradley, Alexandre K Dubé, Ugo Dionne, Christian R Landry

**Affiliations:** Institut de Biologie Intégrative et des Systèmes (IBIS), Université Laval, 1030, Avenue de la Médecine, Québec, QC, Canada G1V 0A6; Regroupement Québécois de Recherche sur la Fonction, l’Ingénierie et les Applications des Protéines, (PROTEO), Université Laval, 1045 Avenue de la Médecine, Québec, QC, Canada G1V 0A6; Centre de recherche en données massives (CRDM), Université Laval, 1065, Avenue de la Médecine, Québec, QC, Canada G1V 0A6; Département de biochimie, microbiologie et bio-informatique, Université Laval, 1045 Avenue de la Médecine, Québec, QC, Canada G1V 0A6; Institut de Biologie Intégrative et des Systèmes (IBIS), Université Laval, 1030, Avenue de la Médecine, Québec, QC, Canada G1V 0A6; Regroupement Québécois de Recherche sur la Fonction, l’Ingénierie et les Applications des Protéines, (PROTEO), Université Laval, 1045 Avenue de la Médecine, Québec, QC, Canada G1V 0A6; Centre de recherche en données massives (CRDM), Université Laval, 1065, Avenue de la Médecine, Québec, QC, Canada G1V 0A6; Département de biochimie, microbiologie et bio-informatique, Université Laval, 1045 Avenue de la Médecine, Québec, QC, Canada G1V 0A6; Département de biologie, Université Laval, 1045 Avenue de la Médecine, Québec, QC, Canada G1V 0A6; Institut de Biologie Intégrative et des Systèmes (IBIS), Université Laval, 1030, Avenue de la Médecine, Québec, QC, Canada G1V 0A6; Regroupement Québécois de Recherche sur la Fonction, l’Ingénierie et les Applications des Protéines, (PROTEO), Université Laval, 1045 Avenue de la Médecine, Québec, QC, Canada G1V 0A6; Centre de recherche en données massives (CRDM), Université Laval, 1065, Avenue de la Médecine, Québec, QC, Canada G1V 0A6; Département de biochimie, microbiologie et bio-informatique, Université Laval, 1045 Avenue de la Médecine, Québec, QC, Canada G1V 0A6; Département de biologie, Université Laval, 1045 Avenue de la Médecine, Québec, QC, Canada G1V 0A6; Institut de Biologie Intégrative et des Systèmes (IBIS), Université Laval, 1030, Avenue de la Médecine, Québec, QC, Canada G1V 0A6; Regroupement Québécois de Recherche sur la Fonction, l’Ingénierie et les Applications des Protéines, (PROTEO), Université Laval, 1045 Avenue de la Médecine, Québec, QC, Canada G1V 0A6; Centre de Recherche du Centre Hospitalier Universitaire (CHU) de Québec, Université Laval, Québec, QC, Canada G1R 2J6; Lunenfeld-Tanenbaum Research Institute, Sinai Health, Toronto, ON, Canada M5G 1X5; Institut de Biologie Intégrative et des Systèmes (IBIS), Université Laval, 1030, Avenue de la Médecine, Québec, QC, Canada G1V 0A6; Regroupement Québécois de Recherche sur la Fonction, l’Ingénierie et les Applications des Protéines, (PROTEO), Université Laval, 1045 Avenue de la Médecine, Québec, QC, Canada G1V 0A6; Centre de recherche en données massives (CRDM), Université Laval, 1065, Avenue de la Médecine, Québec, QC, Canada G1V 0A6; Département de biochimie, microbiologie et bio-informatique, Université Laval, 1045 Avenue de la Médecine, Québec, QC, Canada G1V 0A6; Département de biologie, Université Laval, 1045 Avenue de la Médecine, Québec, QC, Canada G1V 0A6

**Keywords:** SRC homology 3 (SH3) domain, gene duplication, ancestral sequence reconstruction, myosins

## Abstract

Protein–protein interactions (PPIs) drive many cellular processes. Some interactions are directed by Src homology 3 (SH3) domains that bind proline-rich motifs on other proteins. The evolution of the binding specificity of SH3 domains is not completely understood, particularly following gene duplication. Paralogous genes accumulate mutations that can modify protein functions and, for SH3 domains, their binding preferences. Here, we examined how the binding of the SH3 domains of 2 paralogous yeast type I myosins, Myo3 and Myo5, evolved following duplication. We found that the paralogs have subtly different SH3-dependent interaction profiles. However, by swapping SH3 domains between the paralogs and characterizing the SH3 domains freed from their protein context, we find that very few of the differences in interactions, if any, depend on the SH3 domains themselves. We used ancestral sequence reconstruction to resurrect the preduplication SH3 domains and examined, moving back in time, how the binding preference changed. Although the most recent ancestor of the 2 domains had a very similar binding preference as the extant ones, older ancestral domains displayed a gradual loss of interaction with the modern interaction partners when inserted in the extant paralogs. Molecular docking and experimental characterization of the free ancestral domains showed that their affinity with the proline motifs is likely not the cause for this loss of binding. Taken together, our results suggest that a SH3 and its host protein could create intramolecular or allosteric interactions essential for the SH3-dependent PPIs, making domains not functionally equivalent even when they have the same binding specificity.

## Introduction

Protein domains are important structural and functional components of most proteins and often have modular and specific functions (Doolittle 1995). Among these domains are PDZ (PSD95-Dlg-Zo1), Src homology (SH) 2, and SH3 domains (Kuriyan and Cowburn 1997; Castagnoli *et al.* 2004) that direct protein–protein interactions (PPIs) by binding specific short linear motifs present on other proteins. SH3 domains (hereafter referred to as SH3s) have become powerful models to study the evolution of binding affinity and specificity because they are common and involved in many signaling pathways in eukaryotes (Kay *et al.* 2000; Dionne *et al.* 2022). The study of SH3 binding specificity and evolution is also relevant for biomedical applications. Indeed, SH3s bind short linear motifs, and these motifs are often mutated in cancer and are the target of pathogens, allowing them to rewire the signaling pathways of their host (Via *et al.* 2015; Dionne et al. 2022; Mihalič *et al.* 2023). Also, SH3s are found in several oncogenes and essential proteins for signal transduction (Latysheva et al. 2016; Massimino *et al.* 2021). Finally, SH3s can be useful for biotechnological applications by enhancing binding between an enzyme and its substrate and for the assembly of synthetic protein scaffolds (Dueber *et al.* 2009; Park *et al.* 2022).

SH3s are numerous in the budding yeast (*Saccharomyces cerevisiae*) (*n* = 27) and human proteomes (*n* > 300) (Xin *et al.* 2013; Teyra *et al.* 2017), largely as a result of the duplication of the genes that code for their host proteins (Vogel and Chothia 2006). Since the binding specificity of SH3s is key for the function of SH3-containing proteins, substantial effort has been put in place to map the determinants of their binding specificity. SH3-binding motifs most often correspond to PXXP (where X is any amino acid) and fall into canonical groups (Kuriyan and Cowburn 1997; Tonikian *et al.* 2009; Teyra *et al.* 2017). These groups sometimes overlap such that a given SH3 shares binding preference with others. This, in principle, allows different SH3s to bind overlapping sets of proteins. However, it has been suggested that negative selection could act to limit spurious binding with noncognate partners, which would prevent crosstalk between pathways with independent functions (Zarrinpar *et al.* 2003). Several other layers of regulation, including other interaction domains and protein colocalization, therefore combine with motif recognition to direct PPIs and allow a greater specificity (Dionne *et al.* 2022). This was recently illustrated by large-scale PPI-binding efforts that have shown that SH3-directed PPIs *in vivo* also depend on the protein host and the position of the domain in the protein, which was defined as the protein context (Dionne *et al.* 2021). Since there are many mechanisms that contribute to the specificity of PPIs among SH3-containing proteins, one important question is how much do the protein-binding domains themselves contribute to the divergence of PPIs among paralogous proteins?

One powerful way to answer this question is to explore the evolution of the domains that have been preserved after their recent duplication. These domains and their host proteins had the same amino acid sequence at the moment of duplication, which makes it easier to understand how a relatively small number of amino acid changes contribute to a shift in specificity. Among the 27 SH3s present in 18 proteins of the budding yeast are 4 pairs of paralogous domains that originated from a whole-genome duplication (WGD) event 100–150 million years ago (Wolfe and Shields 1997; Marcet-Houben and Gabaldón 2015; Wolfe 2015). Here, we focus on one of these protein pairs, the type I myosins Myo3 and Myo5.

Myo3 and Myo5 localize to actin cortical patches and are involved in endocytosis and exocytosis (Mochida *et al.* 2002). They both contain C-terminal SH3s and have overlapping sets of physical interactions in the Arp2/3 complex (Evangelista *et al.* 2000; Tarassov *et al.* 2008) and in actin polymerization networks (Lechler *et al.* 2000). From a genetics point of view, these 2 genes are at least partially redundant. The single deletion of either *MYO3* or *MYO5* has a slight effect on growth, but a double deletion leads to severe growth and actin cytoskeleton assembly defects (Goodson and Spudich 1995). Myo3 and Myo5 SH3s are a powerful model to study paralogous evolution because their multiple shared PPIs are critical for many cellular processes. The myosin paralogous SH3 domains have 11 different residues out of 59. These differences could have led to significant changes in the SH3-binding preferences because it has been found that a single substitution can lead to PPI disruption (Evangelista *et al.* 2000; Geli *et al.* 2000; Mochida *et al.* 2002; Dionne *et al.* 2021). In addition, they also have multiple known PPIs and a few specific PPIs, 2 for Myo3 and 5 for Myo5 (Szklarczyk *et al.* 2019), which could provide the resolution needed to dissect the divergence of specificity early after duplication.

In this study, we focus on the divergence of PPIs between Myo3 and Myo5 and examine the contribution of their SH3s to this divergence. We first examined the protein interaction network *in vivo* of the 2 paralogous proteins to identify their SH3-dependent PPIs. We then swapped the domains between the 2 paralogs to examine their contribution to PPI divergence. We also used resurrected ancestral SH3s to examine how the 2 domains have diverged since their duplication and pushed further back in time to examine how they have evolved prior to the duplication event. Finally, to examine how the SH3s themselves may have changed independently of the rest of the proteins, we examined the PPI profile of the SH3s *in silico* and free from their protein context.

## Methods

### Sequence analysis

A multiple sequence alignment (MSA) of the paralogs' full-length protein sequence was performed with the MUSCLE v3.8.1551 online tool (Edgar 2004; Madeira *et al.* 2022) using the sequences retrieved from the *Saccharomyces* Genome Database (SGD; Cherry *et al.* 2012).

### Strain construction

Yeast strains used for the DHFR PCA experiments were retrieved from the Yeast Protein Interactome Collection (Tarassov *et al.* 2008) or constructed here and in Dionne *et al.* 2021. Supplementary Table 12 in Supplementary File 1 provides a list of the strain backgrounds and plasmids used to construct the strains. All of the SH3 codon optimized strains (14) were constructed in this study, while WT codon SH3 bait strains (8) were retrieved from Dionne *et al.* (2021). Bait strains were constructed using strain BY4741 (Mata, *his3Δ leu2Δ met15Δ ura3Δ*) according to Tarassov *et al.* (2008) (Supplementary Table 10 in Supplementary File 1). Of the prey strains, 6 were individually reconstructed using BY4742 (Matα, *his3Δ leu2Δ lys2Δ ura3Δ*), and 290 were directly retrieved from the Yeast Protein Interactome Collection (Supplementary Table 11 in Supplementary File 1). Preys of interest were selected based on a previous study (Dionne *et al.* 2021) and manually curated from the *Saccharomyces* Genome Database (Cherry *et al.* 2012). CRISPR-Cas9 genome editing and homologous recombination were used to construct the paralog variants, the motifΔ preys, and the free SH3 constructs (Ryan *et al.* 2016). The appropriate media were used to select for positive clones. NAT (Cedarlane Labs), HYG (Bioshop Canada), and G418 (Bioshop Canada) selections were used at final concentrations of 100, 250, and 200 μg/ml, respectively. The detailed methods are available in Supplementary File 2 and illustrated by Supplementary Fig. 10, a–c.

### DHFR PCA growth conditions

Bait and prey strains were grown on YPD medium [1% yeast extract, 2% tryptone, 2% glucose, and 2% agar (for solid medium)] containing their specific antibiotics. NAT and HYG were used for diploid selection. DHFR PCA selection was done on a synthetic medium (PCA medium, 0.67% yeast nitrogen base without amino acids and without ammonium sulfate, 2% glucose, 2.5% noble agar, drop-out without adenine, methionine, and lysine) containing 200 μg/ml methotrexate (MTX, Bioshop Canada) diluted in dimethyl sulfoxide (DMSO, Bioshop Canada).

### Protein fragment complementation assays and analyses

The DHFR PCA protocol is based on Tarassov *et al.* (2008), and it was published as a visualized protocol (Rochette *et al.* 2015). The principal purpose of DHFR PCA experiments is to assay the interaction between 2 proteins *in vivo*, by the complementation of the fragments of a dihydrofolate reductase (DHFR) that is insensitive to MTX, DHFR F[1,2] and DHFR F[3]. The PCA signal is proportional to the amount of reconstituted DHFR in the cell. The detailed protocol is described in Supplementary File 2. Mating between the baits and preys was performed in random arrays (384 format) on a selective medium resulting in 8 or 10 replicates for each PPI. The arrays were condensed into 1,536 formats and then replicated on MTX medium for the assay. Pictures of the microbial growth at the final time point were acquired and used for the analyses.

For each plate picture, the software Pyphe quantified the colony areas [“pyphe-quantify batch –grid auto_1536 –t 1 –d 3 –s 0.05” (Kamrad *et al.* 2020)]. Raw data from the prey array, diploid selection, and PCA selection are available in Supplementary Data 1. Positions were ignored when there was no growth on the diploid selection or on the prey array. The area values were transformed in log_2_ scale. Then, the background and aberrant data were assessed and removed [3 < log_2_(area) < 13.13, Supplementary Fig. 3a]. A scaling using the maximum and minimum values on each plate transformed the data on a scale between 0 and 1 (PPI score, see equation below).


$$\mathrm{PPI}\;\mathrm{score}=\frac{\mathrm{lo}g_{2}(\mathrm{area})-\mathrm{lo}g_{2}(\min.\;\mathrm{area})}{\mathrm{lo}g_{2}(\max.\;\mathrm{area})-\mathrm{lo}g_{2}(\min.\;\mathrm{area})}$$

**Fig. 1.**
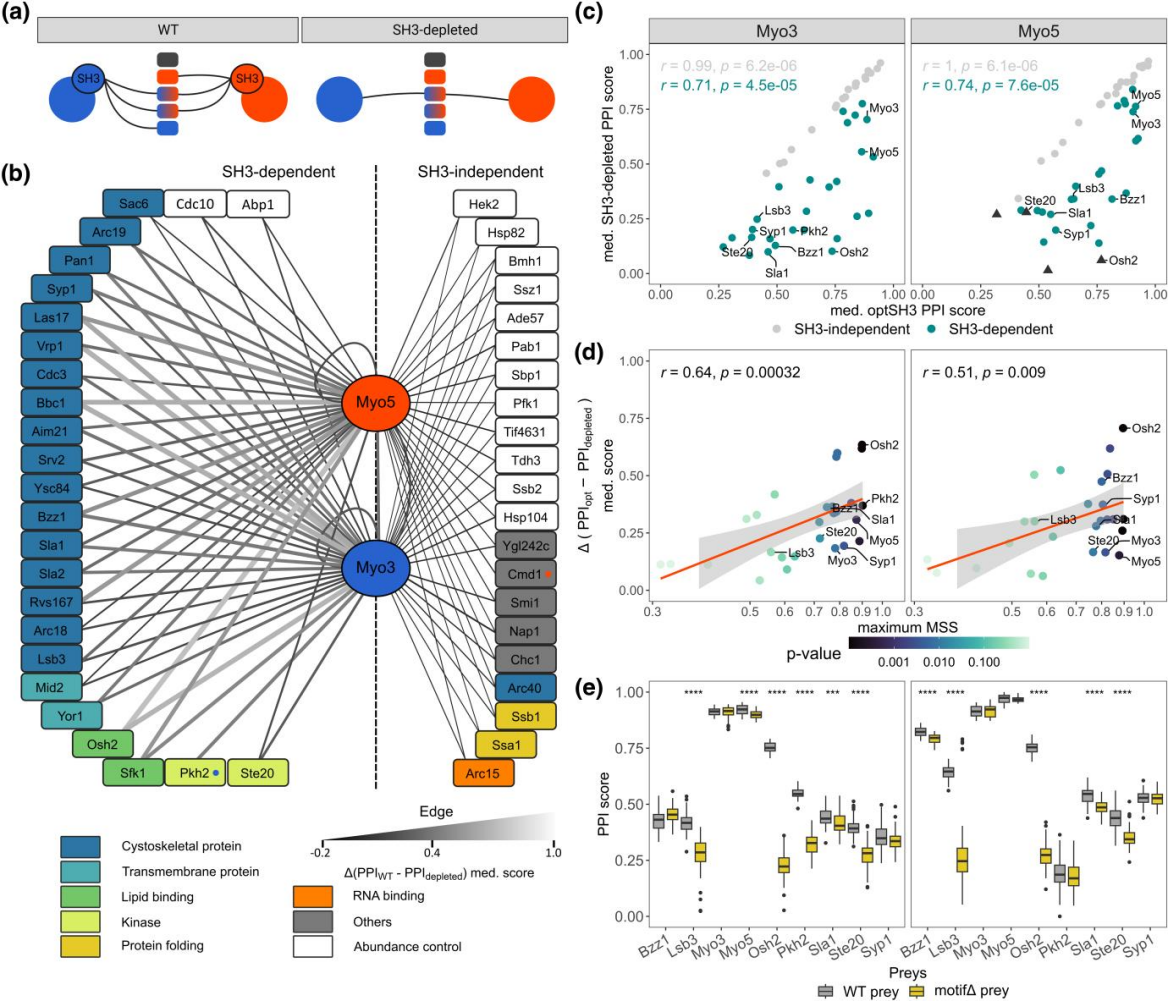
Characterization of the interactomes of Myo3 and Myo5. a) Schematic representation of the experimental design. The baits are shown as circles and preys are shown as rectangles. PPIs are represented with lines, and the color of the prey nodes shows the binding preference of each paralog. The left panel represents the WT PPI network of the paralogs, and the right panel shows the PPI network of the SH3-depleted variants. The comparison between experiments allows us to identify SH3-dependent PPIs. b) Myo3 and Myo5 PPI network. The bait proteins are displayed in the center of the network. The edges show the SH3 contribution to the PPI: Δ(PPI_WT_–PPI_depleted_) median score. The vertical dashed line separates the SH3-dependent, for one or both paralog, and SH3-independent preys as determined by Benjamini–Hochberg-corrected Wilcoxon tests (*P* < 0.05). Paralog-specific partners are indicated by a colored dot in the nodes (Myo3: blue, Myo5: red). Node colors indicate functions (Cherry *et al.* 2012). White nodes are abundance controls. c) Scatter plots comparing the PPI scores of the optSH3 paralog variants with the SH3-depleted paralogs. Myo3 data are shown on the left, and Myo5 data are shown on the right as it is for d) and e). Each data point represents the median score (8 or 10 biological replicates) of an interaction with an individual partner. Triangle-shaped data points are PPIs with fewer replicates (*n* ≤ 2), which prevented us from concluding if they were SH3-dependent. The labeled data points are preys that were used to confirm the proline motif predictions in panel e). d) Scatter plot comparing the Δ(PPI_opt_−PPI_depleted_) median score with the corresponding motif prediction score of the preys (maximum MSS). The color scale represents the *P*-value of the motif predictions. e) PPI scores (48 biological replicates) of the WT preys and motifΔ preys with the extant paralogs. Wilcoxon tests were performed between the extant and the motifΔ preys, and significance levels are shown for each significant comparison (***P ≤ 0.001, ****P ≤ 0.0001).

If there were 2 or fewer biological replicates left after data filtering, the PPI scores were ignored. All the statistical analyses performed on PPI scores are illustrated in the figures or mentioned in the main text. The control group of the statistical tests for the comparison between SH3 variants within one paralog was the optSH3 variant. We chose optSH3 as the control group because ancestral sequences were also codon optimized. Many PPIs were validated in individual liquid PCA assays (Supplementary Fig. 3f). The PPI network was visualized with Cytoscape (Fig. 1b; Shannon *et al.* 2003).

### Comparison of interactome with literature

As a quality control of the data, we compared our dataset with 3 PPI databases, BioGRID (v4.4.209, Stark *et al.* 2006), STRING (v11.5, Szklarczyk *et al.* 2019), and MINT (Licata *et al.* 2012). Also, the BioGRID database (v4.4.209, Stark *et al.* 2006) was used as a reference for PPIs detected previously with different experimental methods. The physical PPIs in both orientations (Myo3 and Myo5 as baits and preys) were retrieved and used for comparison (Supplementary Fig. 2, a–d).

### Proline motif prediction

Position weight matrix (PWM) specificity models for yeast SH3s were obtained from Tonikian *et al.* (2009). With the PWMs, a maximum matrix similarity score (MSS) was computed for each SH3-dependent paralog PPI partner (Dionne *et al.* 2021). The scores are values between 0 and 1, where 1 represents a perfect match to the PWM (Kel *et al.* 2003). For each *k*-mer (*k* = number of PWM columns) in the preys, MSSs were calculated, and the maximum MSS *k*-mer across the whole protein sequence was identified as the predicted binding motif. This procedure was performed for the SH3s of Myo3 and Myo5. The *P*-value of the prediction was assigned by testing if the maximum MSS score for one prey was higher than the MSSs obtained for 10,000 random peptides. An empirical *P*-value of 0.05 indicates that the predicted binding motif had a higher MSS than 95% of the random peptides tested against. Proline motif predictions were used to design 9 motifΔ preys for which 7 were validated experimentally (Fig. 1e). The maximum MSS motif positions are shown in Supplementary Fig. 7b (Brennan 2018).

### Green fluorescent protein strain construction and cytometry fluorescence assay

The green fluorescent protein (GFP) DNA sequence and the HPHNT1 resistance cassette were amplified by PCR for genomic integration (Supplementary File 2). The primers (Supplementary Table 8 in Supplementary File 1) were designed to add 40-bp homology arms at each side of the GFP-HPHNT1 amplicon. The DNA fragments were integrated in the bait strains by homologous recombination at the 3′-end of *MYO3* or *MYO5* replacing DHFR F[1,2] and the NATMX4 resistance cassette. Hygromycin selection was used to select for positive colonies and the genomic integration was validated by PCR and Sanger sequencing.

Validated GFP-tagged strains were grown at 30°C in synthetic medium (SC pH 6 HYG, 0.174% yeast nitrogen base without amino acids and without ammonium sulfate, 2% glucose, 1% succinic acid, 0.6% NaOH, and 0.1% MSG) overnight in triplicates. The cultures were diluted in liquid PCA medium without MTX [0.67% yeast nitrogen base without amino acids and without ammonium sulfate, 2% glucose, drop-out without adenine, and 2% DMSO (Bioshop Canada)] to an optical density (OD) of 0.1 and then grown again until the exponential phase was reached (OD = 0.5–0.7). The cells were diluted to an OD of 0.05 in sterile water, and then the fluorescent intensity of 5,000 cells per replicate was measured on a Guava EasyCyte HT cytometer (blue laser, *λ* = 488 nm).

### Ancestral sequence reconstruction

Ortholog protein sequences of Myo3 and Myo5 were retrieved for fungal species using Ensembl Compara (July 2020, Vilella *et al.* 2009; Supplementary Data 2), ensuring systematic annotations. A MSA of the full-length protein sequences was then produced with MAFFT L-INS-i (v7.453, Katoh *et al.* 2005). Redundant sequences were filtered at a sequence identity threshold of 90%, and heavily gapped positions outside of the SH3 were manually trimmed from the alignment. This processed MSA was used to compute a phylogenetic tree using a maximum-likelihood based approach with IQ-TREE2 (Minh *et al.* 2020; Supplementary Fig. 1a). The best-fitting amino acid substitution model (LG + G4) was searched automatically within IQ-TREE2 and phylogenetic branch support was assessed using the SH-aLRT test (Anisimova *et al.* 2011). The tree is represented in Supplementary Fig. 1a (Letunic and Bork 2021) and respects the known fungal phylogeny (Schoch *et al.* 2020). SH3 domain ancestral sequences were reconstructed with the SH3 domain positions from the entire MSA and the phylogenetic tree using FAST-ML (v3.11, Pupko *et al.* 2000; Ashkenazy *et al.* 2012). This software has the advantage that it reconstructs insertion/deletions (alongside AA substitutions) in the ancestral SH3 domains. Finally, a recent benchmark of MSA softwares for ancestral sequence reconstruction (ASR) revealed the algorithm used here (MAFFT L-INS-i) to be one of the best-performing methods for ASR (Vialle *et al.* 2018). The ASR was performed at every tree node using an LG substitution matrix, maximum-likelihood reconstruction of insertions and deletions, and a joint approach for ASR (“–jointReconstruction yes”). The nodes of interest were chosen using the taxonomic annotations provided by Ensembl Compara. The ancestral sequences at the node of the most recent ancestor for the clades *Saccharomyces* (AncA_1, AncA_2 and AncA_3, 1st to 3rd most likely sequences), *Saccharomycetaceae* (AncC), and *Saccharomycetales* (AncD) were selected. Some species in the WGD clade lost the additional copy of *MYO3*/*MYO5*, so we also selected the WGD node as another ancestral sequence (AncB). The nodes’ ages were estimated with TimeTree (Kumar *et al.* 2017). The boundaries of the Myo3/Myo5 SH3s were determined from the SMART database (V8.0, Letunic *et al.* 2021). Visualizations of the MSAs (Supplementary Fig. 1, d and e) were performed with the R package ggmsa (Zhou *et al.* 2022).

### Molecular docking

The experimental structures of Myo3 (PDB:1RUW) and Myo5 (PDB:1YP5) SH3s were used for molecular docking. The AncD AlphaFold2 (Jumper *et al.* 2021) best-ranked model was also used (Fig. 3c). The structure prediction has a high coverage and pIDDT across the entire structure (Supplementary Data 3). Twenty-eight predicted proline motif structures were computed with AlphaFold2, and the best-ranked model for each motif was used for molecular docking (Supplementary Data 4; Tsaban *et al.* 2022). The molecular docking of the motif-predicted structures on the SH3s was done using Haddock2.4 (Dominguez *et al.* 2003) with parameters optimized for protein–peptide docking (Geng et al. 2017). Ambiguous interaction restraints were defined based on previous work (Janz *et al.* 2007; Supplementary Fig. 8a), and all docking parameters were documented in the run.cns files (Supplementary Data 4). Four hundred structures resulted from each docking run. They were split in clusters according to the Haddock2.4 algorithm. The structures were ranked within each cluster, and the clusters were also ranked by the scoring algorithm. The 10 best structures of each cluster, based on the Haddock2.4 scoring algorithm, were kept for further computations. The global energy of docking structures was minimized with FoldX (“RepairPDB,” Schymkowitz *et al.* 2005) 10 times (Usmanova *et al.* 2018). Then, the interaction energy (Δ*G*) was computed with FoldX (“AnalyseComplex,” Schymkowitz *et al.* 2005) for each energy minimized docking structure. The median Δ*G* across the 10 best structures was computed for each cluster (Supplementary Fig. 8b). All raw and transformed docking data are available in Supplementary Data 4. All protein visualizations were done with ChimeraX (Pettersen *et al.* 2021).

### Motif conservation analysis

Orthologs were retrieved from Ensembl Compara (June 2023, Vilella *et al.* 2009) for all 7 preys where the proline motif had been experimentally validated (Fig. 1e). We then filtered each set of orthologs to only include species that also contain a Myo3/Myo5 ortholog. Each set of orthologs was aligned using MAFFT L-INS-i ( Katoh *et al.* 2005), and the position of the predicted motif in the *S. cerevisiae* copy was mapped to the MSA. Homologous disordered regions in each ortholog were then retrieved using MobiDB to define the boundaries of the disordered regions (Piovesan *et al.* 2023). Finally, the best motif in each region was scored using the Myo3 and Myo5 PWMs, as described previously (see *Methods: Proline motif prediction*). The Lsb3 predicted motif maps to a structured region, and so a ±5 alignment window was taken for the motif search range.

## Results and discussion

### SH3 domains play a major role in the function of Myo3 and Myo5 in vivo

The 2 paralogous genes *MYO3* and *MYO5* originated from the yeast WGD (Wolfe and Shields 1997). The corresponding amino acid sequences are 76% identical. They contain a N-terminal disordered region, a myosin motor, an ATP and actin-binding domain, and a C-terminal SH3 (Supplementary Fig. 7b, Myo3: 1123–1181, Myo5: 1088–1146). Eleven of the 59 residues of their SH3s are different (Supplementary Fig. 1d), which results in very similar structures as shown by the low root-mean-square deviation of atomic positions (RMSD = 0.386 Å, Supplementary Fig. 1b). RMSD < 3Å is only observed between closely related homologous structures (Chothia and Lesk 1986).

We first compared the PPI profiles of Myo3 and Myo5 in *S. cerevisiae*. We used them as baits in a screen with a set of 296 proteins as preys. We measured pairwise PPIs using the dihydrofolate reductase protein-fragment complementation assay (DHFR PCA) (Tarassov *et al.* 2008; Fig. 1a). The preys were selected based on previously detected PPIs and the signaling network of either or both paralogs including 93 known interaction partners (see *Methods*). Proteins that have been shown to interact ubiquitously in DHFR PCA were also included in the assay. We refer to them as abundance controls. These proteins create nonspecific PPIs with most of the yeast proteins, likely through spontaneous DHFR fragment complementation. Thus, they can be used to detect changes in protein abundance (Tarassov *et al.* 2008; Levy *et al.* 2014) as PCA signal correlates with the amount of reconstituted DHFR enzyme (Freschi *et al.* 2013). The more abundant a bait protein is, the stronger the PCA signal generated by the abundance controls will be. We included variants of Myo3 and Myo5, as baits, for which the SH3s were encoded in codon-optimized sequences (optSH3s) for expression in *S. cerevisiae* since we also test codon-optimized ancestral SH3s below. Also, because our goal was to examine the role of the SH3s in the divergence of PPIs, the experiment included SH3-depleted paralog variants for which a short flexible peptide (GGSSGGGG) replaces the SH3s. We used these constructs as baits to determine which PPIs are SH3-dependent (Fig. 1a; Dionne *et al.* 2021).

We detected most of the previously reported PPIs in 3 different PPIs databases (83/135) (Supplementary Fig. 2a; Stark *et al.* 2006; Licata *et al.* 2012; Szklarczyk *et al.* 2019), and the majority of those detected by more than one method (78/95) using the BioGRID as a reference (Stark *et al.* 2006). Excluding the abundance controls for which information is absent from the reference dataset, only one interaction was detected for the first time (Supplementary Fig. 2b). Myo3 and Myo5 share most of their interaction partners (Fig. 1b). Excluding the abundance controls, the paralogs share 32 PPIs and they have one specific PPI each (Myo3: Pkh2, Myo5: Cmd1, Fig. 1b; Supplementary Fig. 4a). We observe significant differences in PPI strength for 13 of the shared ones. However, specific PPIs identified in previous work were not detected in our assay. Broadly, PPI mapping thus confirms the common function of the paralogs in the actin cytoskeleton organization (Goodson and Spudich 1995; Lechler *et al.* 2000) and their functional redundancy. Several PPIs and the quantitative nature of the PCA signal were validated by performing small-scale validation experiments in individual liquid cultures (Spearman, optSH3s *r* = 0.92, *P* = 3.3 × 10^−6^, Supplementary Fig. 3f).

The comparison between the median interaction scores (PPI score, see *Methods*) of the WT codon and the optSH3 reveals very strong correlations for both paralog (Spearman, *r* ≥ 0.99, *P* ≤ 1.6 × 10^−6^), and the optSH3s and WT SH3s PPI scores have similar medians (WT Myo3: 0.764, optMyo3: 0.756, WT Myo5: 0.765, optMyo5: 0.769), suggesting that codon optimization has no effect on the DHFR PCA results. Thus, the data obtained with the optSH3 paralog variants were used as a reference to analyze further experiments testing codon-optimized SH3s. Henceforth, we refer to the WT domains as extant domains (extantSH3s) as we also discuss ancestral domains below. We identified SH3-dependent partners by analyzing DHFR PCA signals of the optSH3 paralog variants compared with the SH3-depleted variants (Fig. 1c). PPIs were classified as SH3-dependent when a significant difference in PPI scores was observed between the SH3-depleted and optSH3 paralog variants (Wilcoxon, Benjamini–Hochberg-corrected *P* < 0.05). We observed for Myo3 25 SH3-dependent PPIs and 21 for Myo5. Excluding the Myo3-specific PPI with Pkh2, the 3 additional SH3-dependent PPIs of Myo3 can be explained by the lack of PCA signal for the SH3-depleted Myo5 variant, which prevents the statistical test from being conclusive (Supplementary Figure 4). Also, Myo3 and Myo5 interact with each other, displaying a modest but significant SH3-dependent property. Some of these SH3 dependencies support the results from previous studies. For example, as we observe here when the entire domain is removed, a previously characterized mutation in the SH3s is known to disrupt SH3-dependent PPIs between Myo3 (W1158S) and Las17 (Evangelista *et al.* 2000), between Myo5 (W1123S) and Bbc1 and between Myo5 and Vrp1 (Geli *et al.* 2000; Mochida *et al.* 2002; Fig. 1b).

We manually reviewed the function of each interaction partner using the SGD (Cherry *et al.* 2012). This analysis revealed that the SH3-dependent partners are mostly involved in the cytoskeleton assembly, whereas the SH3-independent partners are involved in broader cellular functions or are the abundance controls (Fig. 1b). More than half (14/23) of the SH3-independent PPIs involve the abundance controls, which are not expected to be dependent on the bait protein, leaving only 9 specific SH3-independent PPIs. Also, some SH3-independent PPI partners could interact ubiquitously. For example, the abundance control Ssb2 and its paralog, Ssb1, are known to have a similar function as cytosolic chaperons (Werner-Washburne *et al.* 1987; Nelson *et al.* 1992; Fig. 1b), but Ssb1 is not classified as an abundance control. The finding that many of the Myo3 and Myo5 PPIs depend on their SH3s confirms that the SH3s have an important role in the function of these 2 proteins (Geli *et al.* 2000).

To validate our observations on SH3 dependency, the putative proline-rich binding motifs were predicted on the SH3-dependent interaction partners. Predictions were done using a specificity matrix of the preferred binding peptides determined by peptide phage display (Tonikian *et al.* 2009). A motif prediction score [maximum matrix similarity score (MSS)] between 0 and 1 was obtained for each prey by identifying the protein sub-sequence with the strongest match to the Myo3/Myo5 specificity matrix. A positive correlation was observed between the SH3 contribution to the interaction, i.e. the difference of PCA signal between the optSH3 and SH3-depleted protein variants (Δ(PPI_opt_−PPI_SH3-depleted_) median score), and the motif prediction score for both paralogs (Spearman, Myo3 *r* = 0.64 *P* = 3.2 × 10^−4^, Myo5 *r* = 0.51 *P* = 9.0 × 10^−3^, Fig. 1d). This supports our prediction that SH3-dependent PPIs depend on the match of the cognate Myo3 and Myo5 SH3-binding motifs to their interaction partners, although other major factors such as prey abundance could also contribute to variation in PCA signal. To further validate the SH3 and motif dependency of PPIs, we used the same linker sequences as used above to delete the SH3s, but this time to replace the highest scoring motifs on the interaction partners, creating motifΔ preys. We successfully modified 9 of these motifs and measured their impact on PPIs by DHFR PCA (Supplementary Tables 5 and 11 in Supplementary File 1).

A significant decrease in interaction strength for 6 partners of Myo3 and 5 for Myo5 was observed when the top-scoring proline motif was replaced with the linker. SH3-dependent PPIs are therefore most likely dependent on the top-scoring proline motif on the interaction partners (Fig. 1e). Cases where no dependency or partial loss of PPI is observed could be due to redundancy in the motifs as many of the interaction partners contain multiple proline-rich motifs (Supplementary Fig. 7b) or to the contribution of the disordered regions surrounding the motif. These regions are known to be involved in PPIs and to favor multiple binding conformations, as we can hypothesize for Myo3 and Myo5 (Kelil *et al.* 2016; Morris *et al.* 2021). For example, we detected a motif dependency for the interaction of Myo3 with the Myo5 motif but not the reciprocal interaction (Fig. 1e). However, there are many proline motifs in the disordered regions of Myo3 and Myo5 (Supplementary Fig. 7b). One possibility is that the replacement of the proline motif in Myo3 was masked by one of the other proline-rich motifs in proximity, but it would not be the case for the PPI of Myo3-Δmotif with Myo5. Indeed, Myo5 has 5 alternative motifs, and Myo3 has 3 alternative motifs that could compensate for the loss of the maximum scoring motif (Supplementary Fig. 7b). Furthermore, the mechanisms behind the contribution of disordered regions to PPI are also undefined, but the modifications of these regions, as we did by replacing the binding motif, can also affect binding in unexpected ways (Bugge *et al.* 2020).

### SH3-dependent interaction differences between paralogs are not explained by the SH3 contribution to the PPIs

**Fig. 2.**
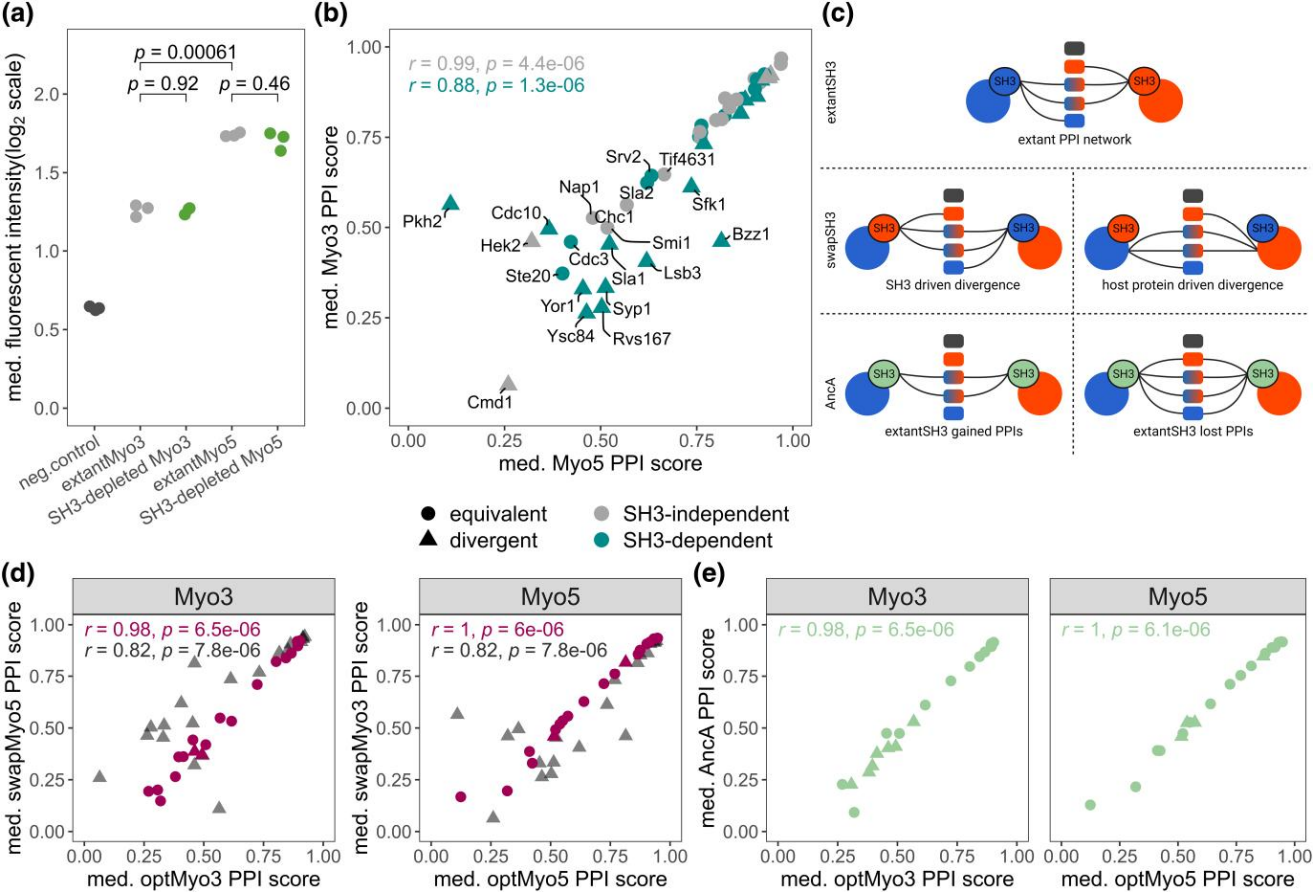
SH3 domains are not sufficient to explain the PPI preferences of the paralogs. a) Expression level of extant paralogs and SH3-depleted protein variants tagged with GFP. The median cell fluorescence signal (arbitrary units) was measured for 5,000 individual cells from 3 independent cultures (individual data point). The negative control is a strain not expressing GFP (strain BY4741). Student's *t*-test *P*-values are shown above each comparison. b) Comparison of the optSH3 variants interaction profiles of Myo3 and Myo5. The colors represent the SH3 dependency of the PPIs. Triangles are divergent PPIs between the paralogs. c) Schematic representation of the expected PPI network of the swapSH3 and AncA paralog variants compared with the extant PPI network under different evolutionary scenarios. The PPIs are represented by lines, and the colors of the nodes show the extant paralog partners. The swapSH3 determines if the contribution of the native SH3 is essential for the paralog-specific PPIs. The PPIs observed with AncA identify which paralog-specific PPI was gained or lost following the duplication event. d) and e) Comparison of the PPIs of Myo3 and Myo5 containing various SH3s. The host protein is shown at the top of each plot. d) The comparisons between the swapSH3 (*y*-axis) and the optSH3 (*x*-axis) in fuschia. The gray data points represent what we would expect if the divergence was SH3-driven, which would allow the swapSH3 to recover all the extant SH3-dependent PPIs and replicate the PPI profiles of the SH3 in their respective paralog context. e) The comparisons of the AncA (*y*-axis) with optMyo3 or optMyo5 (*x*-axis) in both paralogs. Spearman correlation coefficients are shown on the plots.

We compared the Myo3 and Myo5 interaction datasets, and we observed that the Myo5 raw PCA signal is stronger on average than the Myo3 raw PCA signal (Supplementary Fig. 3b, Wilcoxon *P* < 2.2 × 10^−16^). Since DHFR PCA measures the amount of protein complex formed (Remy and Michnick 2007; Freschi *et al.* 2013), differences in binding could in principle result from differences in bait abundance. In order to confirm that this bias is due to protein abundance, we measured protein expression levels using Myo3 and Myo5 GFP-tagged strains (Fig. 2a). The results reveal that Myo5 is slightly more abundant than Myo3 (Fig. 2a, Student's *t*-test, *P* = 6.1 × 10^4^), as reported in public databases (Cherry *et al.* 2012; Breker *et al.* 2013). We also included the SH3-depleted proteins to examine if the deletion of the SH3s affected abundance. Dionne et al. 2021 showed that SH3 depletion had minimal to no effect on abundance by Western blotting for many SH3-containing proteins. We observed the same results here using flow cytometry (Fig. 2a, Student's *t*-test, Myo3 *P* = 0.92, Myo5 *P* = 0.46). Thus, to compare the relative interaction strengths of the 2 paralogs with their preys, we had to ensure that the normalization method to compute the PPI score eliminated the effect of bait abundance on the interaction signal (see *Methods*). Indeed, the median PPI scores of the abundance controls with the paralog variants confirm that the abundance bias was removed by the normalization (Supplementary Fig. 3c, Wilcoxon *P* = 0.96).

We quantified the similarity in binding profiles of Myo3 and Myo5 by directly comparing the normalized PPI scores of the 2 extant proteins. This comparison reveals a strong correlation for SH3-dependent interaction scores (Spearman, *r* = 0.88, *P* = 1.3 × 10^−6^) and an even stronger correlation for SH3-independent ones (Spearman, *r* = 0.99, *P* = 4.4 × 10^−6^, Fig. 2b). However, there remain a few important differences among the SH3-dependent PPIs. For example, Bzz1 is a strong binding partner for Myo5 but a weak one for Myo3, and we observe the opposite pattern for Pkh2 (Fig. 2b). Most of the differences between the 2 paralogs are observed for PPIs with weaker signals. DHFR PCA signal can reach saturation for stronger PPIs (Remy and Michnick 2007), potentially preventing us from detecting divergence for high-intensity binding partners. Furthermore, if the divergence in SH3s is contributing to the divergence of binding, we would expect those to be for cases of weak PPIs as SH3s form weak transient interactions with specific proline motifs (Dalgarno *et al.* 1997; Kay *et al.* 2000). Sequence divergence in the SH3s could thus affect these PPIs most strongly. These results suggest that the difference in binding between Myo3 and Myo5 could potentially be driven by changes in their SH3.

In order to determine whether the SH3s are sufficient to confer PPI specificity to the paralogs, we swapped the SH3s between them (Fig. 2c). A paralogous SH3 inserted in the nonnative paralog is referred to as a swapSH3 paralog variant. If the SH3s are sufficient to confer PPI specificity, swapping the domains will also switch the SH3-dependent PPI profile of the paralogs. Any differences caused by the paralogous SH3s could result from sequence changes following their last common ancestor, resulting in gains or losses of PPI strength for either paralog (Fig. 2c). To differentiate gains and losses of PPIs, the ancestral domain at the last common ancestor node (AncA) was resurrected, and its codon-optimized sequence was also inserted in both paralogs. The ancestral sequence reconstruction was performed using a maximum likelihood-generated phylogeny across fungal Myo3/Myo5 orthologs with Fast-ML (see *Methods*, Pupko *et al.* 2000; Ashkenazy *et al.* 2012; Supplementary Fig. 1a and Data 2). We included only fungal orthologs in the phylogeny because sequence similarity drops as low as 39% between orthologs in this group. Adding more divergent orthologs from outside the fungal kingdom would have added little further insight into the Myo3/Myo5 evolutionary history post duplication. The Myo3/Myo5 tree topology was supported by strong phylogenetic branch supports (aLRT), and all ancestral sequences tested were reconstructed with high posterior probability (Supplementary Fig. 1c). Myo3 and Myo5 SH3 paralogous sequences are mostly conserved, differing in 11/59 positions and we observed that 20/59 positions diverged in at least one ortholog since their most recent common ancestor (Supplementary Fig. 1d). Therefore, the AncA has high sequence identity with both extant SH3s (53/59 Myo3, 54/59 Myo5, Supplementary Fig. 1e). The AncA sequence was reconstructed with high confidence (aLRT = 96.6), and 58/59 positions are predicted with 95% or higher posterior probability (Supplementary Fig. 1c). The insertion of swapSH3 and of AncA in the extant paralogous contexts creates chimeras that could form PPIs with the proteins present in the cell.

Swapping the SH3s and inserting AncA in Myo3 and Myo5 revealed that the interaction profiles of the 2 paralogs are not affected by the difference in the sequence of their paralogous SH3s and their ancestor (Fig. 2, d and e). The paralogs with swapped SH3s have very similar SH3-dependent interaction profiles as their native ones (Fig. 2d, Spearman, Myo3 Myo3 *r* = 0.98 *P* = 6.5 × 10^−6^, Myo5 *r* = 1 *P* = 6 × 10^−6^). Previously, we have found that the PPI intensity between paralogs is different for 13 cases out of 25 SH3-dependent PPIs (Supplementary Fig. 4a). For instance, Pkh2 interacts with Myo3 but not with Myo5, at least in our assays. This specific PPI with Myo3 is SH3-dependent, indicating that the SH3 plays an important role in this PPI. Thus, we expected to detect a similar PPI intensity with Pkh2 when the Myo3 SH3 was inserted in Myo5, but we observed no gain in PPI with the Myo5 swapSH3 variant (Supplementary Fig. 4c). It was previously found that the protein context affects the PPI profiles of SH3 domains (Dionne *et al.* 2021). Yet, in some cases, swapping the SH3s between 2 proteins induced novel specific interactions to the new host protein (Dionne *et al.* 2021). We expected that the effect of the protein context would be reduced with closely related proteins, but, despite the contribution of the SH3 in the Pkh2 PPI and the high sequence similarity between the paralogs, the swapSH3 is not able to bring Myo5 to interact with Pkh2. The same result was obtained with the insertion of AncA in both paralogs (Fig. 2e, Spearman, Myo3 *r* = 0.98 *P* = 6.5 × 10^−6^, Myo5 *r* = 1 *P* = 6.1 × 10^−6^). Overall, these results show that, although different in sequence, the 2 paralogous domains and their last common ancestor do not contribute to differences in binding between Myo3 and Myo5.

### Ancestral SH3 domains progressively lose SH3-dependent PPIs

**Fig. 3.**
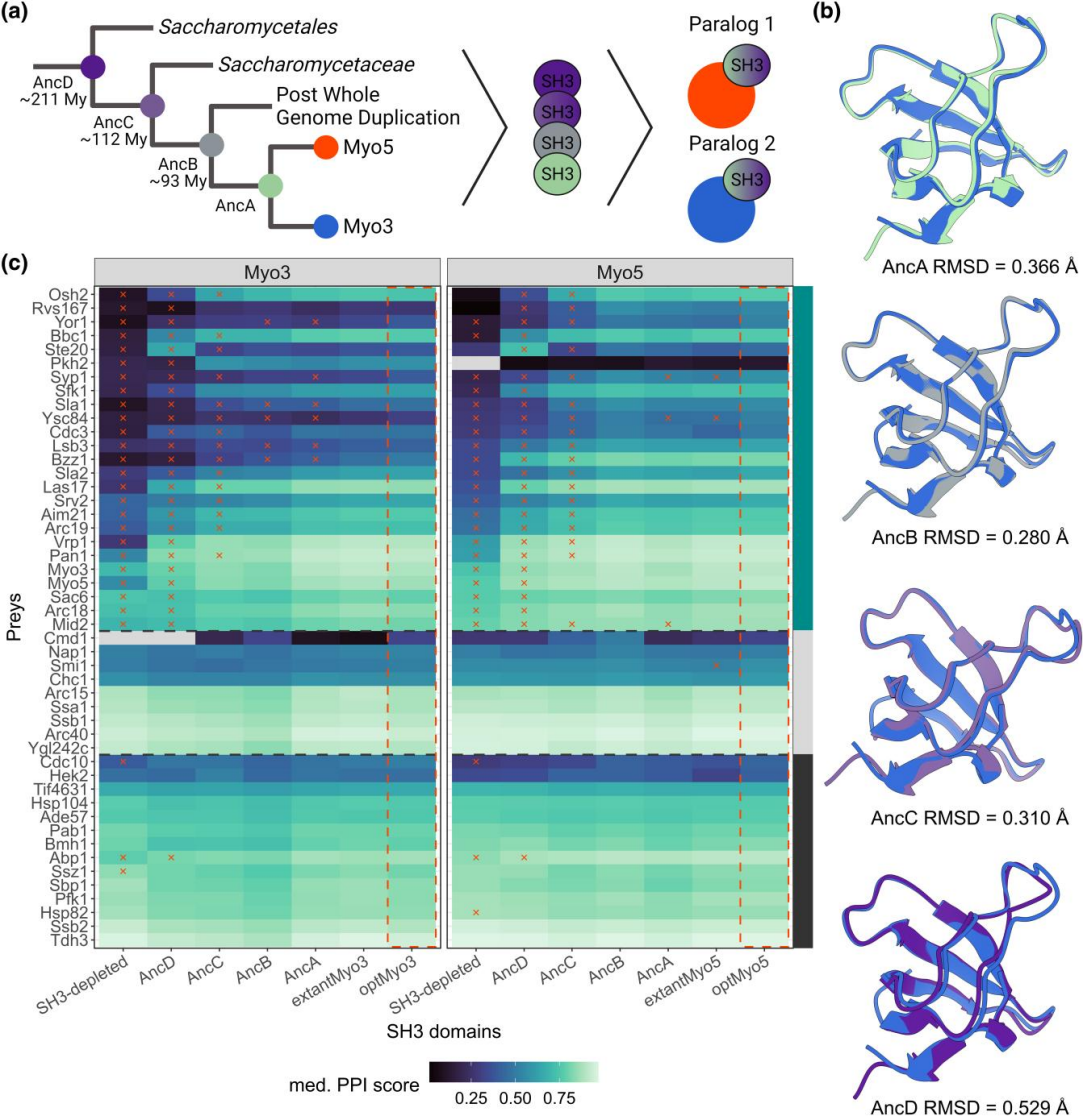
Ancestral SH3 domains affect the PPIs of Myo3 and Myo5. a) Simplified phylogenetic relationship of *MYO3/MYO5* sequences where nodes represent an extant SH3 (blue and red) or an ancestral SH3 that was reconstructed, with an estimation of the time at which these domains existed. b) Superimposed extantMyo3 SH3 structure (blue, PDB:1RUW) and the AlphaFold2-predicted structures of each reconstructed ancestral SH3 showing high structural conservation as estimated by RMSD. c) Heatmap showing the PPIs of SH3 variants inserted in Myo3 and Myo5 with each of their interacting partners (preys, *y*-axis). The preys are ordered by SH3-depleted Myo5 PCA signal and by interaction type with the paralogs (right annotation, cyan: SH3-dependent for one or both paralogs, gray: SH3-independent, black: abundance controls). The tile color corresponds to the median PPI score, and the red x marks correspond to a significant difference (*P* < 0.05, Wilcoxon test, Benjamini–Hochberg correction) in interaction strength compared with the control interaction (dashed red frame). Missing data are shown in gray.

Our results comparing Myo3 and Myo5 PPIs showed that they have quantitatively slightly different interaction profiles, especially for SH3-dependent PPIs (Fig. 2b). However, none of the differences observed appears to be driven by the divergence of the SH3s themselves. Surprised by this, we wondered how robust the PPIs of these 2 paralogs were to the identity of their SH3s. We therefore resurrected more ancestral sequences at speciation nodes (AncB, AncC, and AncD) that predate the most recent common ancestor of Myo3 and Myo5 and examined their PPIs again (Fig. 3a). AncB is the SH3 that was present in the last common ancestor of all the WGD species. Some of these species lost the second copy of the Myo3/Myo5 orthologs. AncC and AncD are, respectively, the last common ancestors for the *Saccharomycetaceae* (family) and *Saccharomycetales* (order) clades. The same method as the one used for AncA was applied to infer AncB, AncC, and AncD domain sequences. Strong topology supports (high aLTR) were obtained even for the oldest node used for the ancestral reconstruction (aLRT, AncB = 85.6, AncC = 89, AncD = 99.9, Supplementary Fig. 1a). However, AncD's posterior probability distribution is slightly lower than the more recent ancestral SH3s, which is expected due to the higher sequence diversity used for the reconstruction (Supplementary Fig. 1c and Data 2). Sequence identity gradually decreases to 55% between the extant Myo3 SH3 and AncD and to 50% for Myo5 SH3 compared with AncD (Supplementary Fig. 1e). To support the ancestral sequence predictions, we used AlphaFold2 to predict the ancestral SH3 3D structures (Fig. 3b; Supplementary Data 3; Jumper *et al.* 2021). The predicted structures show very low RMSD when compared with the extantSH3s, indicating overall structure conservation despite the low sequence identity.

By measuring PPIs of Myo3 and Myo5 containing these ancestral sequences, we observed a general and progressive loss of signal as domains get more ancient, specifically for SH3-dependent PPIs (Fig. 3c). For the most ancient SH3, the PPI network showed a significant interaction loss for its SH3-dependent PPIs compared with the optSH3s (paired-Wilcoxon, Myo3 *P* = 5.5 × 10^−6^, Myo5 *P* = 2.4 × 10^−7^). However, no difference is observed for the SH3-independent median PPI scores across SH3-depleted, AncD and optSH3 variants (Kruskal–Wallis, Myo3 *P* = 0.47, Myo5 *P* = 0.98). The pattern of loss for AncD PPI indicates that the ancestral domain loses PPIs in a similar way to the SH3-depleted paralogs (Fig. 3c). Since SH3-independent PPIs with the abundance controls appear to be preserved, the gradual loss of interaction is unlikely to be caused by protein instability and degradation. To validate this hypothesis, we tagged the paralog variants with GFP and measured protein abundance. Protein abundance was not significantly different compared with the extant paralogs for any of the variants (Supplementary Fig. 3e). Therefore, the results point toward an actual loss of binding by the ancestral domains. An exception to this general tendency is observed for the prey Ste20, which gains interaction with the AncD paralog variants (see below).

Since the SH3s are surrounded by disordered regions containing proline motifs, we explored the hypothesis of intramolecular binding mediated by the ancestral SH3s to explain the loss of PPI with the extant partners. It was shown that AlphaFold2 is able to predict intramolecular binding of a peptide chain containing a SH3 followed by a disordered linker and a proline motif (Tsaban *et al.* 2022). Thus, we used AlphaFold2 to predict the structure of the paralogs carrying the ancestral SH3s (AncA to AncD, Supplementary Data 3; Jumper *et al.* 2021). These analyses did not predict intramolecular binding. In addition, we performed AlphaFold Multimer with Myo3, Myo5, and AncD SH3 domains and their surrounding regions in the paralogs to assess if another proline motif could be favored for the binding of AncD variants (Supplementary Fig. 7b, Myo3: 961–1272, Myo5: 961–1219; Evans *et al.* 2022). However, AncD is predicted to bind the same motifs as the extant SH3s (Supplementary Data 3). Thus, despite the predictions, we are not able to define the role of the surrounding disordered regions in the binding of the SH3s. It is known that AlphaFold2 predictions are less reliable for disordered regions because the algorithm considers coils as unstructured, which can lead to the overestimation of disordered regions (Wilson *et al.* 2022). In our case, this bias in the prediction could prevent us from detecting transient intramolecular or intermolecular binding between a proline motif and the ancestral SH3s; thus, we cannot exclude this mechanism for the loss of interaction of AncD.

We performed an orthology analysis on the SH3-dependent interaction partners of the myosins to examine if binding to the same partners as the *S. cerevisiae* Myo3/Myo5 could have taken place across the clade defined by AncD. We found that most of the interaction partners have orthologs in the species included in AncD clade (Supplementary Fig. 5a). Thus, AncD interaction loss with the extant PPI partners cannot be explained by the absence of their ancestors at the AncD time of existence. We also examined the conservation of the validated proline motifs (Fig. 1e) on PPI partners' orthologs (Supplementary Fig. 5b). We found that proline motifs are present on the orthologs in AncD clade, except for Pkh2. The case of Pkh2 is interesting because we showed that it was a Myo3-specific interaction partner. Pkh2 has a paralog, Pkh1, which was included in the DHFR PCA experiment, but showed no PPI with either myosins. Since Pkh2 binds specifically to Myo3, we could speculate that this is a SH3-dependent interaction gained by Myo3 since the duplication event. This hypothesis is also supported by the motif conservation analysis, because very few proline binding motifs are predicted on the orthologs of Pkh2 outside of the WGD clade, indicating that it was probably not interacting with the ancestral myosin (Supplementary Fig. 5b). Overall, the orthology analysis and the motif conservation analysis suggest that AncD was likely binding to most ancestors of the extant interaction partners.

We investigated the surface properties of the SH3 domains to examine the possibility that the surface outside of the SH3 binding region could contribute to a transient intramolecular conformation with the rest of the protein. We compared hydrophobicity and electrostatic potential of Myo5 SH3 and AncD surfaces using ChimeraX metrics (Supplementary Fig. 6; Laguerre *et al.* 1997; Pettersen *et al.* 2021). The same analyses were performed on Myo3 SH3, and very similar results to Myo5 SH3 were obtained due to the high sequence similarity between the 2 extant SH3s. Only Myo5 SH3 and AncD surface properties are illustrated in Supplementary Fig. 6. Hydrophobicity properties are conserved between the 2 SH3s. However, we observed that a surface patch with negative electrostatic potential on Myo5 SH3 is neutral on AncD (Supplementary Fig. 6). This surface change could potentially affect intramolecular binding and explain our results, but this would require further investigation. We also performed an intramolecular coevolution analysis on the full-length proteins using EVcouplings (v0.2, Hopf *et al.* 2019), but no significant coevolution signal was detected between the SH3 domains and the rest of the proteins (Supplementary Data 6). In short, multiple *in silico* predictions were performed, but none of the approaches allowed us to discern the mechanism behind AncD's loss of binding.

### SH3 variants affinity to proline motifs does not explain the divergence between the paralogous PPIs

The general decrease of binding for ancestral sequences, particularly of AncD, could be caused by a diminished binding affinity of this ancestral domain with the extant binding motifs. Using the binding motif predictions on SH3-dependent preys validated above (Fig. 1, d and e), we performed *in silico* SH3-peptide docking with the ancestral and extant SH3s to test this hypothesis.

**Fig. 4.**
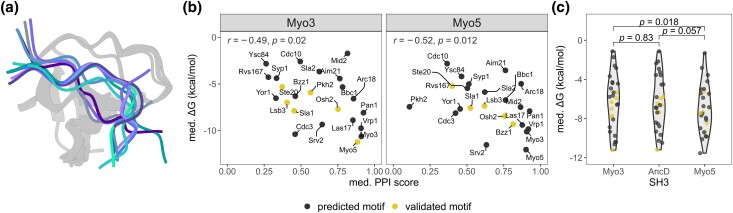
Contribution of SH3 binding to their motifs estimated by molecular docking. a) Superimposed structures of the 10 best-scored docking structures of the Osh2 peptide binding to Myo3 SH3. The peptides show a variety of binding conformations, whereas the SH3 structures remain similar. b) Scatter plots showing the relationship between the computed median Δ*G*s of the docked peptide-SH3 complex and the corresponding PPI scores of the extant paralogs as measured by DHFR PCA. Yellow data points correspond to proteins for which the proline motifs were validated *in vivo* (Fig. 1e). Spearman correlation coefficients (*r*) and *P*-values appear at the top of each plot. c) Violin plot comparing the distribution of median Δ*G* values for each SH3s. Paired Wilcoxon tests *P*-values are labeled on top of the violin plot.

The experimental structures of the extant Myo3 (PDB:1RUW) and Myo5 (PDB:1YP5) SH3s and the predicted AncD structure (Fig. 3b; Supplementary Data 3) were used for computational molecular docking (Geng *et al.* 2017) with peptides corresponding to binding motifs (10 amino acids long) from the preys (Supplementary Table 5 in Supplementary File 1). Nine of these binding motif predictions were used to validate the SH3 dependency (Fig. 1, d and e). Multiple structures were examined each time, and they were sorted into clusters based on RMSD. The clusters and structures were ranked by the docking scoring algorithm (Geng *et al.* 2017). Next, a global energy minimization step was applied to the best structures of each cluster and the SH3–peptide interaction energies were computed [Δ*G* (kcal/mol)] (Schymkowitz *et al.* 2005). In 43% of the docking peptide–SH3 combinations, the best-ranked cluster included the lowest Δ*G* structures (Myo3: 8/28, Myo5: 15/28, AncD: 13/28). Additionally, we observed various conformations in the docked peptide structures even if these structures are grouped in the same cluster (Fig. 4a). We thus computed the median Δ*G* of the best structures of each cluster to consider this variability (see *Methods : Molecular Docking*). Using the median Δ*G* to compare the clusters, we validated that the ranking of the clusters is a good indicator of the binding energy. Indeed, the first rank cluster shows a lower distribution of median Δ*G* compared with clusters with a lower rank for each SH3 (Supplementary Fig. 8b). Thus, we continued the analysis with the median Δ*G* of the top cluster of each docking as a proxy for the affinity.

We observe significant correlations between the median Δ*G*s and the PPI scores obtained by DHFR PCA for both paralogs (Spearman, Myo3 *r* = −0.49 *P* = 0.02, Myo5 *r* = −0.52 *P* = 0.012) (Fig. 4b). This result supports the binding motif predictions illustrated in Fig. 1d given that the peptides used for structural modeling derive from our binding motif predictions. To examine if the paralogous SH3s have different binding affinities to the proline motifs, we compared the median Δ*G*s between Myo3 and Myo5 SH3s (Fig. 4c). This comparison suggests that Myo5 SH3 binds globally more strongly to the proline motifs (predicted and validated) than Myo3 SH3 (paired-Wilcoxon, *P* = 0.018). In addition, the comparisons between AncD and the extantSH3s median Δ*G*s do not explain the data collected *in vivo*. Indeed, there is no significant decrease in binding affinity computed for AncD compared with the extantSH3s (paired-Wilcoxon, Myo3 *P* = 0.83, Myo5 *P* = 0.057), while a general decrease in binding is detected *in vivo* for the AncD variants. We note however that in these comparisons, AncD appears to have median Δ*G*s more similar to that of Myo3 SH3 than of Myo5 SH3, although the differences are not significant. Thus, even if the docking reveals a globally stronger binding of the proline motifs to Myo5 SH3 compared with Myo3 SH3, it does not explain the differences in the binding profile of the extant SH3s *in vivo*. The inconclusive molecular docking results and the fact that they are not in agreement with the *in vivo* experiments led us to seek validation with additional *in vivo* experiments.

The conflicting results between the PCA experiment and the molecular docking do not allow us to conclude as to whether the SH3 affinity for the proline motifs is one of the main factors shaping the differences in PPIs of Myo3 and Myo5 nor to explain the loss of interaction shown with the replacement of native SH3 by the AncD variant in the paralogs. Another hypothesis that could explain the PCA results is that the PPIs of the ancestral SH3 with the preys are reduced because the SH3 also interacts (intramolecular interaction or allosterically) with its host protein in a way that decreases its ability to bind. The context in which the SH3 is found may therefore be a strong determinant of its ability to bind their recognition motif, as recently shown by Dionne *et al.* (2021) for more distantly related SH3s. We therefore performed experiments with the SH3s free of their protein context.

### Free SH3s lose paralog-specific PPIs pattern, highlighting the role of protein context on SH3-dependent PPIs

**Fig. 5.**
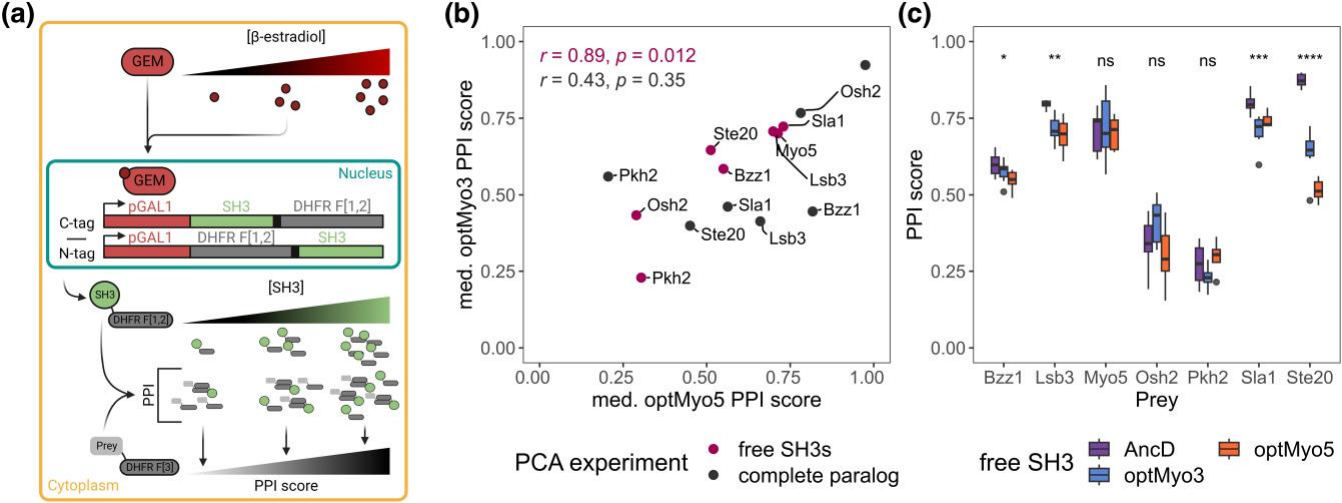
Characterization of free SH3 binding profiles. a) Schematic representation of the DHFR PCA experiment using free SH3s with an inducible promoter. The transcription factor GEM binds to β-estradiol and induces the expression of the SH3 coding sequence fused with the DHFR F[1,2] via the *GAL1* promoter. The expression of the construct increases with the amount of β-estradiol, leading to an increase of the DHFR PCA signal. b) Scatter plot comparing the PPI profile of the free paralogous SH3s and that of the complete paralogs (entire proteins with the optSH3s). Spearman correlation coefficients (*r*) and *P*-values for each experiment are shown. c) Boxplot of the PPI scores for several preys (*x*-axis) with 3 SH3 variants. Kruskal–Wallis tests compare the scores among the 3 variants for each prey. *P*-value significance levels are labeled at the top of each group (ns: *P* > 0.05, *P ≤ 0.05, **P ≤ 0.01, ***P ≤ 0.001, ****P ≤ 0.0001).

The SH3s were fused directly to a DHFR fragment to measure PPIs, which has been done with other reporter proteins to identify binding motifs (Wu *et al.* 2007; Tonikian *et al.* 2009). All the SH3 variants described previously were expressed by fusing them to the DHFR F[1,2], and we tested PPIs with the preys for which we successfully identified a binding motif (*n* = 7). The free SH3s were all tagged at either terminus to test the effect of the position of the DHFR F[1,2] on the SH3s PPIs. The SH3 coding sequences were inserted at a genomic locus regulated by the *GAL1* promoter, and the expression was under the control of a synthetic circuit induced by β-estradiol (Aranda-Díaz *et al.* 2017; Fig. 5a). This allowed us to test PPIs at various bait expression levels to ensure that we measure PPIs below PCA signal saturation. Analysis of the data shows that the DHFR F[1,2] C-tag empty construct causes less background growth than the DHFR F[1,2] N-tag empty construct (Wilcoxon, *P* < 2.2 × 10^−16^), so we focused on the DHFR F[1,2] C-tag constructs.

We tested multiple expression levels by changing β-estradiol concentrations, and we observed that median PPI scores reached saturation in a prey-dependent manner. To compare the different SH3 variants, we chose a β-estradiol concentration in the range of maximum sensitivity of the assay, below saturation (see *Methods*, Supplementary Fig. 9b). The difference of median PPI score Δ(WT preys−motifΔ preys) in the complete paralogs measured above and the free SH3s show a significant correlation for optMyo3 (Spearman, *r* = 0.89, *P* = 0.012) and the same tendency for optMyo5 (Spearman, *r* = 0.64, *P* = 0.14, Supplementary Fig. 9c), suggesting that the free SH3s bind the preys in a similar manner as the SH3s in their host protein context.

The comparison of the median PPI scores between free optMyo3 and free optMyo5 shows a strong significant correlation (Spearman, *r* = 0.89 *P* = 0.012, Fig. 5b). We observed no bias in interaction strength toward optMyo5 SH3 compared with optMyo3 SH3 when they were expressed isolated from the proteins (Fig. 5b). This supports our results of the swapping experiment that showed that the affinity of the Myo5 SH3 for the preys tested is not higher than the affinity of the Myo3 SH3. However, we tested only 7 preys in this experiment, which reduces the resolution to observe divergence. Even if we find that the free paralogous domains generate similar PPIs, the free SH3s did not recreate the Myo3 and Myo5 SH3-dependent PPI profiles. For example, the PPIs of the paralogs with Osh2 were very strong and were highly affected by the SH3 depletion (Fig. 3c), but the free SH3s were not able to generate as strong PPIs (Fig. 5b). This could be explained by the competition happening between the free SH3 and the extant paralogs for the binding of their target peptides in those strains. The strains expressing the free SH3s are also expressing the 2 gene copies of the WT paralogs.

As observed previously, the SH3s showed differences in the PPI scores, but, unlike in the experiments with the entire proteins, we did not detect a systematic decrease in PPI scores of the free AncD compared with the free optSH3s (Fig. 5c). In some cases, free AncD showed stronger PPIs than the free optSH3s. Even if it seems that the N-tag free AncD loses PPIs, the statistical test is not significant (Supplementary Fig. 9d, Kruskal–Wallis, *P* = 0.44), probably because of the limited number of preys included in the experiment and the generally low signal for this construct (Supplementary Fig. 9d). Likewise, there was no apparent decrease in PCA signal for the C-tag free AncD (Supplementary Fig. 9d, Kruskal–Wallis, *P* = 0.27). This validates the docking results, which predicted that the binding affinity of AncD would be similar to those of the extant domains.

An additional finding we wanted to confirm with these experiments was the stronger interaction between Ste20 and AncD paralog variants when the complete paralogs were considered. We identified the binding motif mediating this PPI (Supplementary Fig. 7c), and we observed a stronger interaction with the free AncD compared with the optSH3s (Wilcoxon, *P* = 1.6 × 10^−4^, Fig. 5c). However, it is difficult to fully rationalize this result as molecular docking does not predict a higher affinity between AncD and Ste20 motif than with the extant SH3s (Fig. 4c). It is possible that AncD and Ste20 do not interact as a typical SH3-peptide interaction; thus, the docking structure restraints used would not be appropriate to study AncD-Ste20 interaction.

Overall, while ancestral SH3s tend to lose PPIs when introduced in the extant protein, they do maintain an ability to interact with their partners when expressed alone. It is well established that SH3 domains have an intrinsic binding specificity that is encoded in their sequence (Teyra *et al.* 2017; Brown *et al.* 2018). However, our results suggest that the ability of the domain to bind motifs on other proteins is also regulated by its host protein via intramolecular interactions or through allostery. Inserting an ancestral sequence into an extant protein could disrupt the regulatory mechanism, leading to the general loss of PPIs despite the fact that the domains themselves maintain their ability to bind. This type of regulation implies that coevolution takes place between an SH3 and the rest of its protein such that the intrinsic binding specificity of a domain is not, alone, sufficient to dictate the specificity of the protein.

## Conclusion

We compared the interaction profiles of 2 recently evolved paralogous proteins, Myo3 and Myo5, that contain SH3s to examine if sequence divergence of the domains contributed to the divergence in binding to other proteins *in vivo*. Compared with the more than 1-billion year-old origin of the SH3 structural family, these 2 paralogous domains have recently diverged (Alvarez-Carreño *et al.* 2021). Our results show that Myo3 and Myo5 have largely overlapping sets of interaction partners, but some of their PPIs, which we showed to be SH3-dependent, are biased for one paralog or the other. To test if the SH3 domains are causing these differences in interactions, we swapped the 2 domains between Myo3 and Myo5. This experiment showed that the 2 domains are functionally equivalent since they had the same PPI profiles. The equivalent function of the 2 paralogous SH3s was also confirmed by inserting in the extant paralogs the ancestral SH3 of their most recent common ancestor. Again, the interaction profiles of the 2 paralogs were not affected.

To examine further the evolution of the SH3s, we looked at more ancient, resurrected domains. We found that in general, Myo3 and Myo5 lose their SH3-dependent PPIs when their SH3s were replaced with older ancestral sequences. However, molecular docking showed that the affinity of the most ancient domain reconstructed, AncD, for the proline motifs is not lower than the one of the extant SH3s. Experiments with the SH3 domains isolated from their host proteins revealed similar results. This suggests that although the SH3s may have maintained their binding specificity for millions of years, the way they interact (via intramolecular interactions or through allostery) with their host proteins may have changed. It was previously shown that the modification of a protein domain affects the function of another domain present in the host protein (Cho *et al.* 2011; Zhang *et al.* 2023). Thus, allosteric or intramolecular interactions between the host proteins, and its SH3 could be disrupted when inserting the ancestral domains into extant proteins, leading to weakened PPIs. Our results thus strengthen the postulate that protein domains are not isolated features of proteins (Melero *et al.* 2014; Vishwanath *et al.* 2018) and that evolution is shaped by coevolution between the domains and the rest of the protein.

## Data Availability

Strains and plasmids are available upon request. Supplementary File 1 contains Supplementary Tables 1–12. Supplementary Tables 1–4 contain (solid and liquid) results from all the DHFR PCA experiments described. Supplementary Table 5 contains the proline motif prediction information. Supplementary Table 6 contains the processed flow cytometry data. Supplementary Table 7 contains a summary of the molecular docking *in silico* experiments. Supplementary Tables 8 and 9 contain, respectively, the oligonucleotides and the SH3 DNA sequences. Supplementary Tables 10 and 11 describe, respectively, the bait and prey strains used in this study. Supplementary Table 12 describes all reagents and resources used in this paper. Supplementary File 2 contains additional details on the methods. Supplementary File 1 and 2 and Figs. 1–10, raw data for the DHFR PCA, AlphaFold2 structure predictions, molecular docking, detailed data from the phylogeny, motif orthology analyses, and myosins sequence coevolution are available on Dryad at: https://doi.org/10.5061/dryad.sj3tx968m (Supplementary Data 1–6) (Lemieux *et al.* 2023). Code used to analyze the raw data and to generate all figures can be found at https://github.com/Landrylab/Lemieux_et_al2023.
